# Further evidence supporting the role of *GTDC1* in glycine metabolism and neurodevelopmental disorders

**DOI:** 10.1038/s41431-024-01603-0

**Published:** 2024-04-11

**Authors:** Edoardo Errichiello, Mauro Lecca, Chiara Vantaggiato, Zoraide Motta, Nicoletta Zanotta, Claudio Zucca, Sara Bertuzzo, Luciano Piubelli, Loredano Pollegioni, Maria Clara Bonaglia

**Affiliations:** 1 Unit of Medical Genetics, Department of Molecular Medicine, University of Pavia, Pavia, Italy; 2 Neurogenetics Research Center, IRCCS Mondino Foundation, Pavia, Italy; 3 Laboratory of Molecular Biology, IRCCS E. Medea, Bosisio Parini, Lecco, Italy; 4 Department of Biotechnology and Life Sciences, University of Insubria, Varese, Italy; 5 Unit of Clinical Neurophysiology and Epilepsy Centre, IRCCS E. Medea, Bosisio Parini, Lecco, Italy; 6 Laboratory of Cytogenetics, IRCCS E. Medea, Bosisio Parini, Lecco, Italy

**Keywords:** Functional genomics, Gene expression

## Abstract

Copy number variants (CNVs) represent the genetic cause of about 15–20% of neurodevelopmental disorders (NDDs). We identified a ~67 kb de novo intragenic deletion on chromosome 2q22.3 in a female individual showing a developmental encephalopathy characterised by epilepsy, severe intellectual disability, speech delay, microcephaly, and thin corpus callosum with facial dysmorphisms. The microdeletion involved exons 5-6 of *GTDC1*, encoding a putative glycosyltransferase, whose expression is particularly enriched in the nervous system. In a previous study, a balanced de novo translocation encompassing *GTDC1* was reported in a male child with global developmental delay and delayed speech and language development. Based on these premises, we explored the transcriptomic profile of our proband to evaluate the functional consequences of the novel *GTDC1* de novo intragenic deletion in relation to the observed neurodevelopmental phenotype. RNA-seq on the proband’s lymphoblastoid cell line (LCL) showed expression changes of glycine/serine and cytokine/chemokine signalling pathways, which are related to neurodevelopment and epileptogenesis. Subsequent analysis by ELISA (enzyme-linked immunosorbent assay) and HPLC (high-performance liquid chromatography) revealed increased levels of glycine in the proband’s LCL and serum compared to matched controls. Given that an increased level of glycine has been observed in the plasma samples of individuals with Rett syndrome, a condition sharing epilepsy, microcephaly, and intellectual disability with our proband, we proposed that the *GTDC1* downregulation is implicated in neurodevelopmental impairment by altering glycine metabolism. Furthermore, our findings expanded the phenotypic spectrum of the novel *GTDC1*-related condition, including microcephaly and epilepsy among relevant clinical features.

## Introduction

Neurodevelopmental disorders (NDDs) impact brain development and function resulting in a wide range of neurological and psychiatric manifestations. These include, but are not limited to, developmental delay/intellectual disability (DD/ID), autism spectrum disorders (ASD), and epilepsy, often associated with microcephaly and various neuroimaging findings, such as corpus callosum and white matter abnormalities. Two or more of such entities may co-occur in the same individual, suggesting that multiple neural circuits are impacted during both pre- and postnatal brain development [1, 2].

The spectrum of genetic alterations associated with NDDs ranges from single nucleotide variants (SNVs) to large chromosomal events [3]. Between these two extremes, structural variants (SVs) greater than 50 bp, referred to as copy number variants (CNVs), have been recognised as an important contributor to NDDs [4–7]. Current approaches to identifying CNVs include chromosomal microarray analysis (CMA), gene‐specific exon‐level arrays, CNV detection from exome sequencing (ES) data and genome sequencing (GS); CMA is still recommended as a first-tier diagnostic test for NDDs, reaching a detection yield around 15–20% [8]. The advent of NGS technology and large-scale studies greatly contributed to the identification of thousands of NDD-associated genes, although a plethora of molecular pathways and loci still await discovery. Furthermore, multiomics approaches, computational models and bioinformatics network analysis have emerged in recent years as a promising strategy to uncover the aberrant functional effects of genomic variations implicated in NDDs [9–11].

By using an integrated omics approach, we investigated the functional consequences of a de novo intragenic deletion of *GTDC1* (MIM *610165), encoding a putative glycosyltransferase, which was detected through routine CMA testing in a female individual with developmental encephalopathy including ID, epilepsy, microcephaly, speech delay, and facial dysmorphisms. To date, disruption of *GTDC1*, as a consequence of a de novo chromosome translocation t(2;8), was reported in only one individual with developmental and speech/language delay [12]. Here we expanded the previously described phenotype and provided further insights on the potential functional consequences of *GTDC1* disruption. Our and previous findings point to *GTDC1* as a novel disease gene involved in neurodevelopment, which should be included in the routine diagnostic workup of NDDs.

## Samples and methods

### Clinical phenotype

The proband is the second child of healthy non-consanguineous Italian parents with unremarkable family history. She was born at full term by caesarean section. Birth weight was 3400 g. Early motor milestones were delayed: she sat at the age of 12 months and reached walking autonomy at 24 months, with frequent loss of balance. Speech and language development were also delayed: she uttered her first words at 3 years, and she was able to construct the simplest sentences at the age of 6. At the age of 8, the first epileptic seizures were suspected, characterised by motor arrest, eyelid myoclonus and non-responsiveness with daily frequency. During an EEG recording at that age atypical absences seizures were documented. EEG background activity was irregularly organised with low amplitude and an excess of widespread rapid rhythms. Asynchronous diffuse bilateral polyspike-waves discharges, prevalently over the posterior regions, were recorded. Intermittent light stimulation triggered the discharges and ictal phenomena. At this age physical examination revealed severe microcephaly (OFC: 46 cm; −4.6 SD) and craniofacial dysmorphisms including long face, hypertelorism, upslanted palpebral fissures, short philtrum, high palate, and prognathism. At Stanford-Binet test, the IQ was 31. In addition to microcephaly, brain MRI showed a thin corpus callosum. Anti-seizure treatment was started with Valproic Acid (VPA). At the age of 20, due to the prolonged seizure-free interval, VPA was gradually tapered but a significant behaviour worsening, characterised by aggression and insomnia, was noted, which was solved by increasing the VPA dose. At the last clinical evaluation (36 years), she was seizure-free and still undergoing treatment with VPA. She currently shows sporadic lipothymic-syncopal episodes often related to microcytic anaemia; an echocardiogram documented mitral valve prolapse with mild insufficiency.

### Chromosomal microarray analysis (CMA) and quantitative PCR (qPCR)

Proband and parental DNA was extracted from venous blood with standard protocols.

CMA was performed on proband’s DNA using an Agilent Human Genome CGH + SNP Microarray Kit 4 × 180 k (G4890A) and subsequently 1x1M (G4447A). Data analysis was performed with the Agilent Cytogenomics v.5.2.0.20 software. All nucleotide positions refer to the GRCh38/hg38 human genome assembly.

Quantitative PCR (qPCR) assays were performed on DNA from the proband and his parents to investigate the de novo or inherited origin of the CNVs, using SYBR Green, and analysed on an ABI PRISM 7900HT sequence detection system (Applied Biosystems, Foster City, CA).

**Fig. 1:**
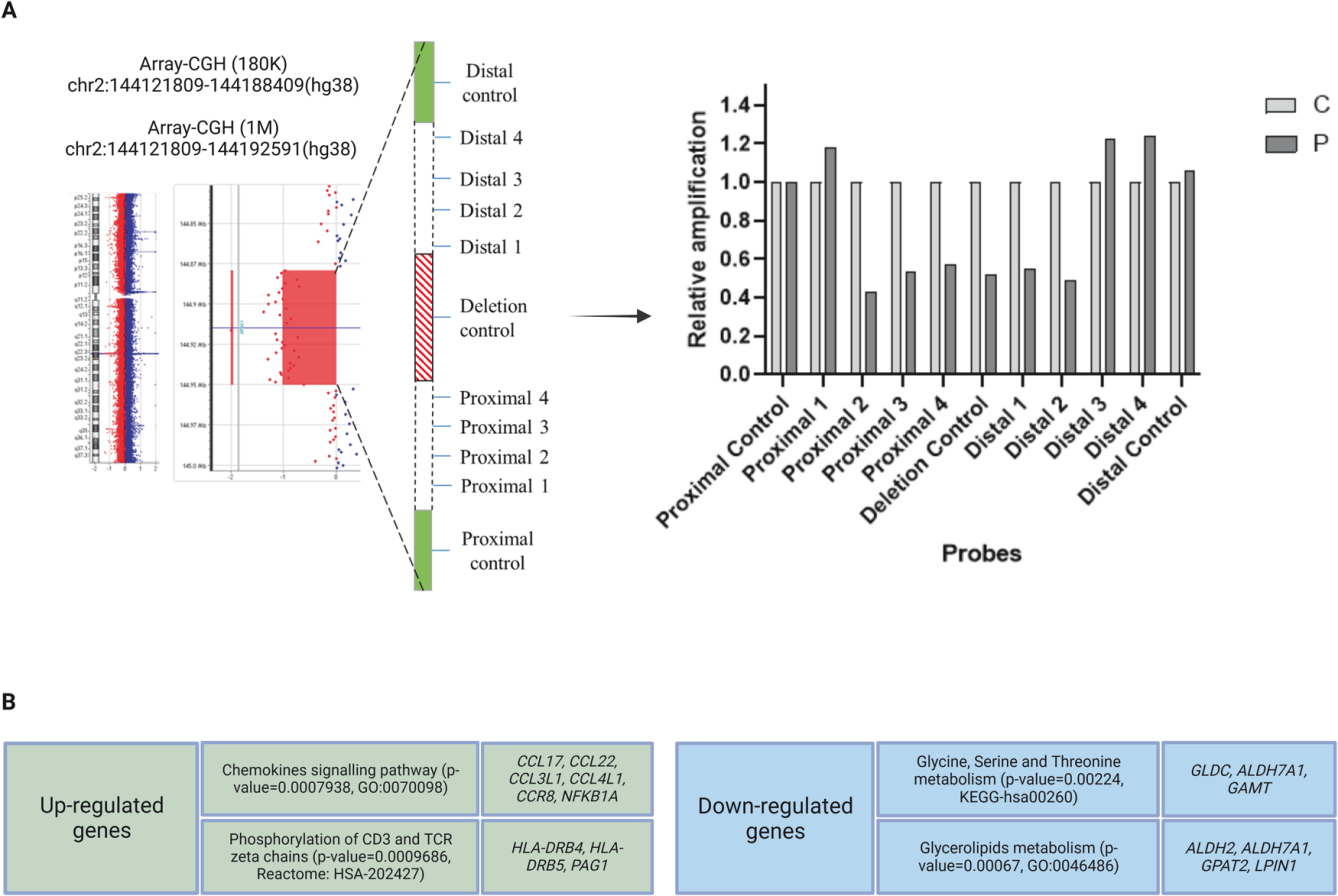
The 2q22.3 microdeletion involving exons 5 and 6 of *GTDC1* and RNA-seq findings in the proband’s lymphoblastoid cell line. **A** Array-CGH (180 K and 1 M platforms) profile showing the de novo 2q22.3 microdeletion removing exons 5 and 6 of *GTDC1*. Further breakpoint analysis was performed by quantitative PCR (qPCR) with probes spanning the deleted genomic region (Proximal 1–4 and Distal 1–4); two additional probes in the non-deleted proximal and distal regions (Proximal/Distal control) and one probe in the centre of microdeletion (Deletion control) were included as controls. Normalisation of qPCR data was performed by using the Proximal control probe. This approach allowed to refine the deletion between chromosomal positions (hg38) chr2:144,119,297 (Proximal 1 probe) and chr2:144,194,675 (Distal 3 probe). P proband, C control. **B** Pathway enrichment analysis by different tools (ToppGene, GeneNetwork, Enrichr, Ingenuity Pathway Analysis, GeneGlobe) highlighted up-regulation of the chemokine signalling pathway and downregulation of glycine/serine/threonine metabolism pathway. More details are available in Supplementary Tables S2 and S3. The figure was created with BioRender (https://www.biorender.com/).

Breakpoint refinement on the proband’s DNA was achieved by qPCR using primer pairs spanning the deleted region (proximal/distal 1-4 probes); two additional probes in the non-deleted proximal and distal regions (proximal/distal control) and one probe in the centre of the microdeletion (deletion control) were included as controls (Fig. 1A and Supplementary Table S1). The qPCR reactions were carried out with the PowerUp^TM^ SYBR^TM^ Green Master Mix (Applied BiosystemsTM, Foster City, California, US) and analysed on a QuantStudio 5 Real-Time PCR System (Thermo Fisher Scientific). All experiments were performed in triplicate, and relative expression was calculated through the 2^-ΔΔCT^ method. The analysis of TADs (Topologically Associating Domains) was performed with 3D Genome Browser (http://3dgenome.fsm.northwestern.edu).

### Trio-based exome sequencing (trio-ES) and bioinformatic analysis

ES was carried out on blood DNA samples of the proband and parents (trio) by using the Human Core Exome kit (Twist Bioscience, South San Francisco, CA, US) on a NovaSeq 6000 System (Illumina, San Diego, CA, US). After reads alignment, variant calling, annotation, and removal of possible artefacts was done as previously reported [13]. Variant filtering was based on the presumed inheritance patterns, list enrichment analysis by using the GeneNetwork, ToppGene and Enrichr tools, phenotype ontology terms (HPO) and in silico panels (PanelApp) selected on the basis of the proband’s clinical records. Variants were classified according to the ACMG/AMP and ACGS guidelines [14, 15].

### Transcriptome sequencing analysis (RNA-seq)

Total RNA was extracted from the B-lymphoblastoid cell line (LCL) of the proband (~1.5 × 10^6^ cells) and a control LCL matched for sex and age (#GM05380; Coriell Institute for Medical Research, Camden, NJ, US) using standard protocols. RNA-seq was performed at BGI Genomics (Yantian District, Shenzhen, China) using the DNBseq platform. cDNA libraries were sequenced with 100 base pairs (bp) paired end (PE) reads and alignment was performed to the GRCh38 reference genome with HISAT2.

### Differential gene expression (DEG) and pathway enrichment analysis

The expression level for each gene was calculated by RSEM, while NOIseq algorithm was used to detect differentially expressed genes (DEGs) in the proband’s LCL compared to control (#GM05380; Coriell Institute for Medical Research, Camden, NJ, US). Low-expressed genes, defined as genes having FPKM < 3 (Fragments Per Kilobase of transcript per Million mapped reads), were filtered out. Genes having a fold change absolute value of log2-Ratio ≥1 and a posterior probability ≥0.8 were considered as differentially expressed. Pathway enrichment analysis was performed by integrating different bioinformatic tools (ToppGene, GeneNetwork, Enrichr, Ingenuity Pathway Analysis, and GeneGlobe) and top enriched pathways were selected for further investigations.

### Quantitative reverse transcription PCR (RT-qPCR)

RT-qPCR was used to confirm the results of the RNA-seq analysis as well as for checking expression levels of *GTDC1* and nearby genes *ZEB2* and *ARHGAP15*. *GAPDH* was used as housekeeping gene, whereas the cDNA deriving from the #GM05380 control cell line as reference sample. cDNA specific primers were designed in exon-exon junctions to avoid undesired gDNA amplification by using ENSEMBL Genome Browser (https://www.ensembl.org/) (Supplementary Table S1). PCR reactions were carried out on a QuantStudio 5 Real-Time PCR System (ThermoFisher Scientific).

### Enzyme-linked immunosorbent assay (ELISA)

Glycine concentration in the proband’s LCL (~1 × 10^6^ cells) and culture supernatant was measured by using the Glycine Assay kit (MAK261; Sigma-Aldrich, St. Louis, MO, US). Interleukin 10 (IL-10) levels in the LCL’s supernatant were detected with the Human IL-10 CatchPoint® SimpleStep ELISA® kit (ab229436, Abcam, Cambridge, UK). Briefly, cells were lysed in the specific Assay Buffer, centrifuged to remove insoluble material, and incubated with enzymes and reaction reagents. Fluorescence emitted by the chromogen dyes was quantified on Fluoroskan microplate reader (Ascent FL, Thermo Fisher Scientific) at 544/590 nm. After background subtraction, the fluorescence of each sample was normalised to protein concentration, previously determined with BCA Protein Assay kit (ThermoFisher Scientific).

### Serum collection and enantiomeric high-performance liquid chromatography (HPLC) analysis

HPLC analysis was performed on serum, LCL and cell culture supernatant. Withdrawal of venous blood of the proband and twelve age and gender-matched unrelated healthy individuals was performed in the morning (8:00–10:00 a.m., after a fasting night) in BD Vacutainer™ SST™ II Advances tubes (Becton Dickinson, Franklin Lakes, NJ, USA) including clot activator and gel for serum separation. Tubes were coded and serum separation was performed by centrifugation. Sera were immediately frozen and stored at −80 °C.

All the chemicals and reagents used in HPLC analysis were purchased from Merck Life Sciences (Darmstadt, Germany), except of methanol, acetonitrile, and tetrahydrofuran (Honeywell International, Seelze, Germany). Serum proteins were removed by addition of 90% HPLC grade methanol followed by vortexing for 3 min at room temperature. Precipitated proteins were eliminated by centrifugation at 16,000 × *g* for 15 min at 4 °C. Supernatants were dried at 55 °C, suspended in 0.2 M trichloroacetic acid and analysed. Lymphoblastoid cells (~5 × 10^6^) were homogenised in 0.1 mL of 0.2 M TCA, sonicated (3 cycles, 10 s each), centrifuged at 16,000 × *g* for 20 min at 4 °C and the supernatants were collected for the analysis. Culture medium samples were added of HCl (25 mM final concentration) and centrifuged at 39,000 × *g* for 30 min at 4 °C.

Ten µL of each sample were neutralised with NaOH and subjected to pre-column derivatization with o-phthalaldehyde and N-acetyl-L-cysteine. Separation of the enantiomers of the amino acids was carried out by reversed-phase HPLC on a Symmetry C8 column (Waters S.p.A., Sesto San Giovanni, MI, Italy) using a HPLC PU-2089 System (Jasco Europe, Cremella, LC, Italy) equipped with a fluorescence detector (344/443 nm, gain 100X). Separation was performed under isocratic conditions in 0.1 M sodium acetate 1% tetrahydrofuran pH 6.2 as mobile phase at 1 mL/min. All investigated amino acids were detected in a single run of 60 min. A washing step with 0.05 M sodium acetate buffer, 47% acetonitrile and 3% tetrahydrofuran pH 6.2, was performed after each run. The identification and quantifications of all amino acids was based on retention times and calibration curves obtained with external standards. Peak identity of D-enantiomers was confirmed by selective degradation by RgDAAO M213R variant [16]: samples were treated with 10 μg of enzyme, incubated at 30 °C for 4 h before derivatization and HPLC analyses. Data obtained from LCL were normalised to total protein content as determined by standard Bradford method.

### Statistical analysis

Student’s *t* test for unpaired variables (two-tailed) and one way ANOVA or two-way ANOVA followed by Dunnett’s, Tukey’s, Welch’s or Sidak’s multiple comparisons tests were performed using GraphPad Prism version 9.3.0 for Windows (GraphPad Software, San Diego, California, US). Results are reported as mean ± SEM; *p*-values less than 0.05 were considered significant. Individual *p*-values are indicated in the graphs (*p < 0.05; **p < 0.01; ***p < 0.001; ***p < 0.0001).

## Results

### Identification of *GTDC1* intragenic deletion

Array-CGH analysis in the proband revealed a de novo copy number loss of 66.6 kb at 2q22.3, involving exons 5-6 (out of 12) of *GTDC1* (RefSeq NM_001376312.2) (Fig. 1A). No other rare copy number changes were detected. Due to lack of sufficient information to interpret the pathogenicity of the variant and based on the constraint metrics of *GTDC1* in gnomAD v2.1.1 (pLI: 0.00; LOEUF: 1.18; pHaplo: 0.12), the CNV was interpreted as a variant of unknown significance (VUS) according to the ACMG classification guidelines [14, 17]. Subsequent trio-based ES did not identify other potentially relevant variants that could explain the proband’s phenotype, especially in known epilepsy-, microcephaly- and ID-associated genes.

A combination of high-resolution array-CGH (1 M platform) and qPCR analysis allowed to refine the deletion boundaries between chromosomal positions (hg38) chr2:144,119,297 (Proximal 1 probe) and chr2:144,194,675 (Distal 3 probe) (Fig. 1A and Supplementary Table S1). Although the peculiar genomic architecture of the region, highly enriched with repeated elements, did not allow the precise breakpoint cloning, we were able to pinpoint the (proximal) 114 bp-L2b LINE element (chr2:144,119,375-144,119,488) and the (distal) 199 bp-MIRc SINE element (chr2:144,194,201-144,194,399) as likely mediators of the chromosomal rearrangement.

Targeted expression analysis by RT-qPCR in the proband’s LCL showed reduced expression of *GTDC1* at about 50% as compared to the control LCL (#GM05380) (Supplementary Fig. S1a), suggesting that the intragenic deletion exerted a loss-of-function effect likely triggering nonsense-mediated mRNA decay (NMD). No mRNA expression changes of both neighbouring genes, *ZEB2* and *ARHGAP15* (Supplementary Fig. S1b), which are contained in the same Topologically Associating Domain (TAD), were observed in the proband’s LCL (as confirmed by RNA-seq).

### Functional studies

#### GTDC1 deletion is associated with altered expression of glycine/serine and cytokine/chemokine signalling pathways

RNA-seq analysis identified 206 differentially expressed genes in the proband’s LCL, of whom 74 upregulated and 132 downregulated, including *GTDC1* (Fig. 1B, Supplementary Tables S2 and  S3).

Pathway enrichment analysis was performed by merging outputs obtained from different tools (ToppGene, GeneNetwork, Enrichr, Ingenuity Pathway Analysis, and GeneGlobe). Top enriched pathways, i.e., those prioritised (*p* < 0.05) by at least two tools, were considered as potential candidates and underwent more careful evaluation. The most significantly upregulated biological pathways were “chemokine-mediated signalling pathway” (*p* = 0.00007938; GO:0070098; top enriched genes: *CCL17, CCL22, CCL3L1, CCL4L1, CCR8*, and *NFKBIA*), and “phosphorylation of CD3 and TCR zeta (ζ) chains” (*p* = 0.00009686; Reactome-HSA-202427; top enriched genes: *HLA-DRB4*, *HLA-DRB5*, and *PAG1*). Conversely, “glycine, serine and threonine metabolism” (*p* = 0.00224; KEGG-hsa00260; top enriched genes: *GLDC*, *GAMT* and *ALDH7A1*) and “glycerolipid metabolism” (*p* = 0.00067; KEGG: map00561, GO:0046486; top enriched genes: *ALDH2*, *ALDH7A1*, *GPAT2*, and *LPIN1*) were the most significantly downregulated pathways. ES excluded the presence of deleterious variants in the top enriched genes that could potentially cause biased expression changes. Among these pathways, chemokine-mediated signalling and glycine/serine/threonine metabolism appeared the most coherent with our proband’s phenotype according to the literature data. In fact, altered glycine and D-serine levels have been related to the development of kindling epileptogenesis in mice [18]. In humans, serine deficiency is associated with severe neurological abnormalities including developmental delay, intellectual disability, microcephaly and seizures [19], while several studies detected low levels of glycine and D-serine in the plasma and cerebrospinal fluid of individuals with schizophrenia [20, 21]. The top downregulated enriched genes in this pathway are key players of neurodevelopment, since biallelic variants of *GLDC*, *GAMT*, and *ALDH7A1* are respectively associated with glycine encephalopathy (GCE; MIM #605899), cerebral creatine deficiency syndrome 2 (CCDS2; MIM #612736) and pyridoxine-dependent epilepsy (EPD; MIM #266100). At the level of single gene analysis, we observed downregulation of *SOCS3*, a suppressor of interleukin-6 (IL-6) signalling, which modulates neuronal development, differentiation, and survival [22]. According to its biological function, dysregulation of IL-6 is implicated in various cognitive dysfunctions, and individuals with high concentrations of circulating IL-6 are at higher risk to develop global cognitive decline, schizophrenia, major depression, and bipolar disorder [23, 24]. Another downregulated gene, *ESAM*, has been recently associated with a rare neurodevelopmental phenotype characterised by DD/ID, epilepsy, absent or severely delayed speech, ventriculomegaly, thin corpus callosum, variable microcephaly and intracranial haemorrhage, which already manifest in the antenatal period in individuals with biallelic loss-of-function variants that completely abrogate gene expression [25].

RNA-seq findings were validated by ELISA and HPLC on different sample types (LCL, cell culture supernatant, and blood serum) of the proband and matched controls, as described below.

#### GTDC1 deletion induces an increase in glycine levels in the proband’s LCL and serum

**Fig. 2:**
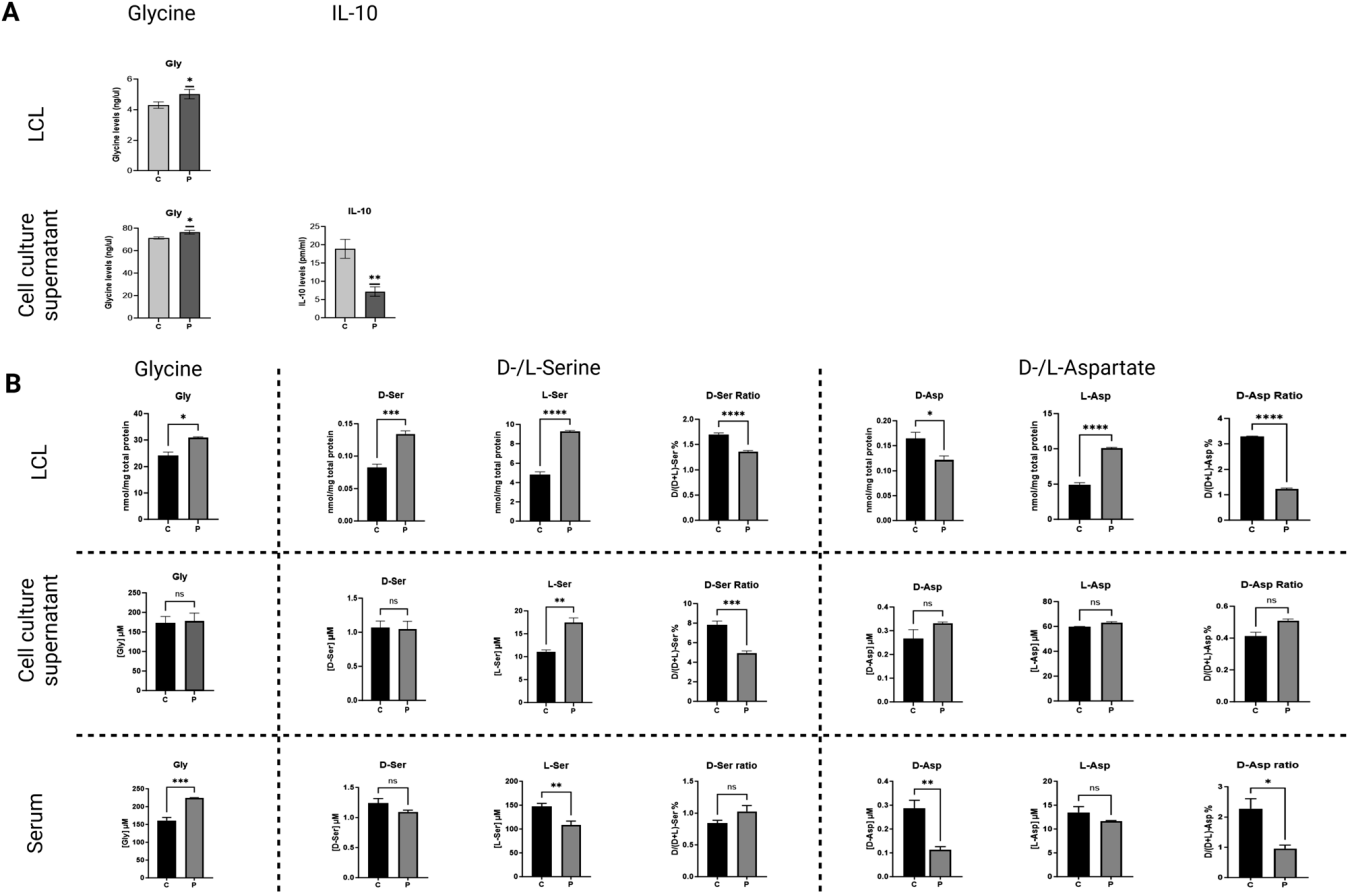
ELISA and enantiomeric HPLC analysis of selected amino acids in the proband and matched control samples. **A** Levels of glycine and IL-10 evaluated by ELISA in the proband’s lymphoblastoid cell line (LCL) and cell culture supernatant. **B** HPLC dosage of glycine, D- and L-serine and D- and L-aspartate in LCL, cell culture supernatant, and blood serum collected from the proband and twelve age and gender-matched unrelated healthy individuals as controls. Data obtained from LCL were normalised to total protein content. C control, Fm fresh medium, P proband, ns not statistically significant. Experiments were performed at least in triplicate. Statistically significant *p*-values are expressed as *p < 0.05, **p < 0.01, ***p < 0.001, and ****p < 0.0001, respectively.

The ELISA test detected increased levels of glycine in the proband’s LCL and cell culture supernatant compared to the control (#GM05380) (Fig. 2A). In addition, reduced levels of interleukin-10 (IL-10) were detected in the proband’s cell culture supernatant (Fig. 2A). To validate these results, glycine and D- and L-serine levels in LCLs, cell culture supernatants and sera of the patient and control, were quantified by HPLC [26]. Aspartate (D- and L-enantiomers) was also evaluated due to its emerging role in influencing N-methyl-D-aspartate receptor (NMDAR)-mediated transmission and neurodevelopment [27, 28]. The HPLC analysis confirmed increased levels of glycine in the proband’s LCL and serum compared to matched controls (Fig. 2B). Furthermore, HPLC detected a significantly increased concentration of both D- and L-serine enantiomers in the proband’s LCL, as well as of L-serine in the supernatant, whereas a decrease in the serum (Fig. 2B). Regarding aspartate, the D-enantiomer was reduced in the proband’s LCL and serum, whereas the L-enantiomer was highly increased in the LCL (Fig. 2B). In this regard, previous evidence demonstrated that D-aspartate was decreased in the dorsolateral prefrontal cortex of individuals affected by schizophrenia [29].

## Discussion

In this study we investigated a female patient with a severe neurodevelopmental disorder and symptomatic epilepsy carrying a de novo microdeletion involving exons 5 and 6 of *GTDC1*, encoding the glycosyltransferase-like domain-containing protein 1 (UniProt Q4AE62), which is highly expressed in the nervous system, especially in the cerebral cortex. Previous experiments showed that disruption of *GTDC1* expression induced defects of proliferation, maturation, and glycosylation in neural progenitor cells (NPCs) and neurons derived from pluripotent stem cells (iPSCs) of a subject carrying a t(2;8) involving *GTDC1*, as well as in knocked-down human embryonic stem cells (ESCs) [12]. Moreover, knock-down of *gtdc1* in zebrafish resulted in neurodevelopmental defects, including microcephalic head, as we observed in our proband.

The link between *GTDC1* and altered neurodevelopment is intriguing and supported by several studies showing that glycosylation finely tunes a myriad of neural functions, such as neurite outgrowth, axon guidance, synaptogenesis, membrane excitability, neurotransmission, and neuroinflammation [30–34]. Furthermore, alterations of glycosylation-related genes (“glycogenes”), especially glycosyltransferases, have been associated with congenital disorders of glycosylation (CDGs), a large group of over 100 rare multisystemic diseases caused by defects in the synthesis of glycans and clinically characterised by mild to severe metabolic defects and a wide range of neurodevelopmental abnormalities, such as ID, epilepsy, and microcephaly [35].

A reduction in *GTDC1* levels, as we observed in our proband, was found in another individual with signs of neurodevelopmental disorder [12]. Theoretically, these findings might suggest haploinsufficiency as a pathogenic mechanism underlying the neurodevelopmental phenotype observed in both individuals, although this seems not to be the case if considering the gnomAD v2.1.1 constraint metrics of *GTDC1* (pLI: 0.00; LOEUF: 1.28; pHaplo: 0.12). Although a meticulous biochemical dissection of the function exerted by the *GTDC1*-encoded protein is mandatory for firm conclusions, it might be speculated that *GTDC1* disruption may exert a trans-dominant negative effect by affecting potential dimerisation or activity of the wild-type enzyme or eventually other associating partners. Otherwise, decreased *GTDC1* expression may reflect in even slightly altered glycosylation processes that might, however, exert wide deleterious effects, especially in bodily districts where glycosylation is considered particularly critical for maintaining physiological cellular functions, such as nervous system.

**Fig. 3:**
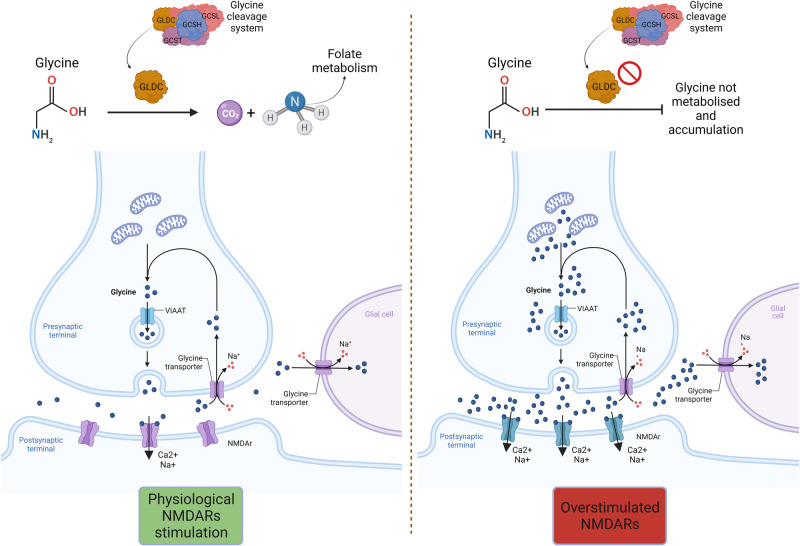
Overstimulation of NMDA receptors due to *GLDC* downregulation and glycine accumulation. In the mitochondrial glycine cleavage system (GCS), composed of four enzymes (encoded by *GCSH*, *GLDC*, *AMT*, and DLD) glycine decarboxylase/GLDC converts glycine into CO_2_ and NH_3_. The reduced *GLDC* expression observed in the proband’s LCL may be reflected in the accumulation of unmetabolised glycine in the cytosol of pre-synaptic neurons, leading to increased release of glycine in the synaptic space and overstimulation the NMDA receptors on the surface of post-synaptic neurons. The figure was created with BioRender (https://www.biorender.com/).

RNA-seq experiments revealed expression changes in glycine/serine and chemokine-mediated signalling pathways in our proband’s LCL. Glycine and serine are biosynthetically linked amino acids in that serine is converted into glycine by the serine hydroxymethyltransferase enzyme (SHMT). The increased levels of glycine we detected in the proband’s LCL and serum by ELISA and HPLC is intriguing, since glycine metabolism has a crucial role in the CNS, being one of the two physiological co-agonists, together with D-serine, of NMDARs involved in synaptic plasticity, neurodevelopment, and neurodegeneration [36]. Accordingly, altered glycine metabolism is implicated in various neurodevelopmental defects [37–40]. Furthermore, increased levels of glycine and serine were detected in the plasma samples of individuals with Rett syndrome (RTT; MIM #312750), a condition including epilepsy, microcephaly and DD/ID among cardinal clinical features [41]. *GLDC*, encoding the glycine decarboxylase, one of the four proteins forming the mitochondrial glycine cleavage system (GCS), and *GAMT*, encoding the guanidinoacetate N-methyltransferase, were among the top downregulated genes in the RNA-seq analysis. Biallelic variants of *GLDC* cause glycine encephalopathy (GCE; MIM #605899), also known as nonketotic hyperglycinemia (NKH), which is clinically characterised by epilepsy, DD/ID, microcephaly, cerebral malformations, such as hypoplasia of the corpus callosum, and typical laboratory finding of hyperglycinemia and hyperglycinuria. Therefore, we speculated that the *GLDC* downregulation observed in our proband may lead to an accumulation of unmetabolized glycine, contributing to an hyperactivation of the post-synaptic NMDARs (Fig. 3). This data is particularly significant considering the epileptic phenotype of our proband. Indeed, it is well known that NMDARs belong to a family of ionotropic glutamate receptors that play essential roles in excitatory neurotransmission and synaptic plasticity in the mammalian CNS [40]. Furthermore, several studies suggest that NMDARs contribute to the genesis and spread of the abnormal paroxysmal discharges, neuron injury and inflammation, and thus may participate in epileptogenesis [40]. Furthermore, glycine level affects L-serine one and the latter amino acid is the precursor of D-serine [42, 43]: the observed increase in proband’s LCL D-serine level could further induce NMDA receptor hyperactivation. On the other hand, GAMT deficiency leads to creatine depletion and guanidinoacetate (GAA) accumulation in brain, which may in turn interfere with neuronal γ-aminobutyric acid (GABA) receptors type A and cause seizures, DD/ID, prominent speech delay, autistic/hyperactive behavioural disorders, and various types of pyramidal and/or extra-pyramidal manifestations in humans [44].

Like glycine and serine metabolism, chemokine-mediated signalling pathway also plays a crucial role in CNS homeostasis by exerting neuroprotective and reparative functions and modulating synaptic transmission, blood brain barrier (BBB) activity and neuroinflammation [45]. Increased levels of pro-inflammatory cytokines (e.g., IL-6 and TNF-α) have been associated with an augmented risk for microcephaly in preterm newborns [46, 47]. Furthermore, aberrant expression of cytokines and chemokines together with their receptors, such as downregulation of IL-10, has been reported in individuals with hippocampal sclerosis and focal cortical dysplasia [48]. In mouse models, it has been shown that IL-10 suppresses the production of pro-inflammatory cytokines/chemokines and inflammasome activation, thus its downregulation might confer decreased anti-inflammatory state and predispose to epileptic seizures [49]. In this regard, our data demonstrated significantly lower levels of IL-10 in the proband’s LCL supernatant compared to the control sample. Interestingly, IL-10 has been demonstrated to modulate cellular response (e.g., in terms of cytosolic calcium concentration) induced by NMDAR activation [50], suggesting an interplay between glycine-induced NMDAR overstimulation and increased pro-inflammatory cytokine levels.

A major limitation of this study is that RNA-seq experiments were performed on the proband’s LCL, which could not properly reflect the expression changes in the CNS. Nevertheless, previous evidence showed that LCLs exhibit broad isoform sharing with brain tissues and therefore can be considered a highly suitable cell source for RNA-seq in NDDs [51]. Furthermore, *GTDC1* expression is adequately detectable in both whole blood and EBV-transformed lymphocytes, according to GTEx and literature data [52]. Therefore, we considered the proband’s LCL as a reliable biological source for mRNA analysis, although expression changes as compared to the nervous system cannot be completely ruled out.

In conclusion, the individual described in this study is the second with altered *GTDC1* expression associated with severe developmental delay. However, unlike the clinical picture of the first reported individual [12], our proband shows microcephaly and symptomatic epileptic syndrome, broadening the phenotypic spectrum of the novel *GTDC1*-related condition.

Although further investigations are needed to reinforce the genotype-phenotype correlation, we propose that *GTDC1* disruption may alter crucial neurodevelopmental processes as a consequence of modifications affecting the glycine metabolism (thus the NMDAR signalling) and the physiological balance between pro- and anti-inflammatory cytokines/chemokines.

### Supplementary information

Supplemental material

## Data Availability

All data generated or analysed during this study are included in this published article [and its supplementary information files].
