# Supplementary material for: Further evidence supporting the role of GTDC1 in glycine metabolism and neurodevelopmental disorders
Source: Eur J Hum Genet. 2024 Apr 11;32(8):920–7. doi: 10.1038/s41431-024-01603-0 (PMC11291697; doi:10.1038/s41431-024-01603-0)
Supplement: Supplementary file 1 — Supplemental material [file 41431_2024_1603_MOESM1_ESM.docx]

**Supplementary Material**

**
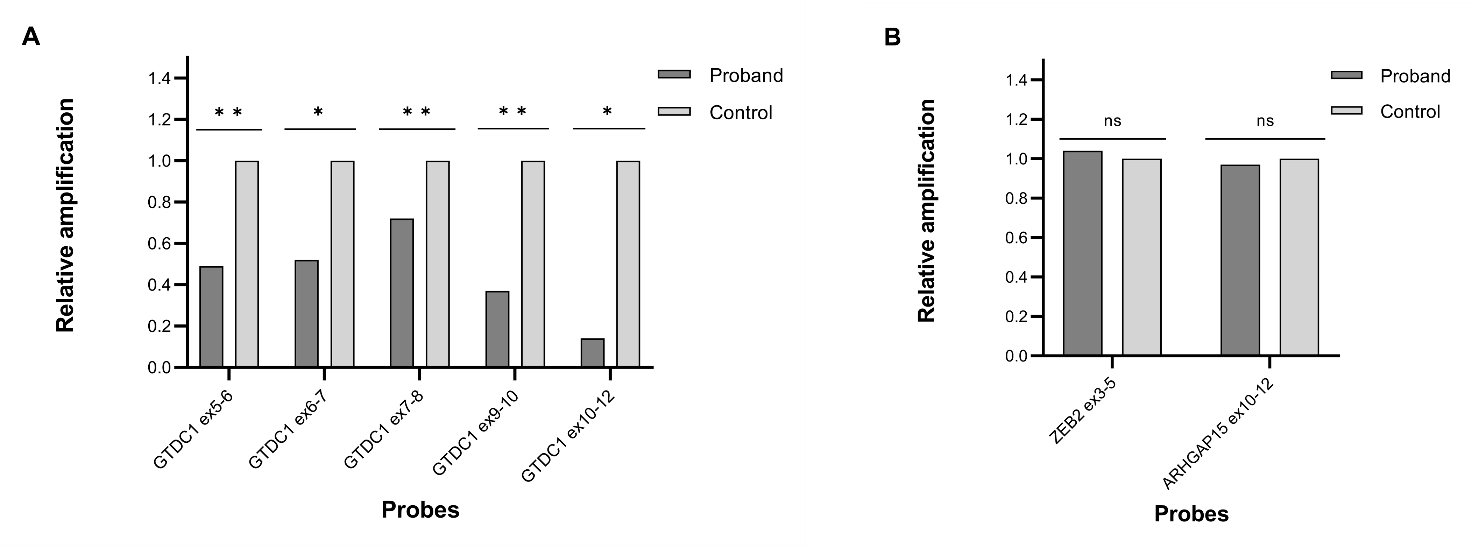
**

**
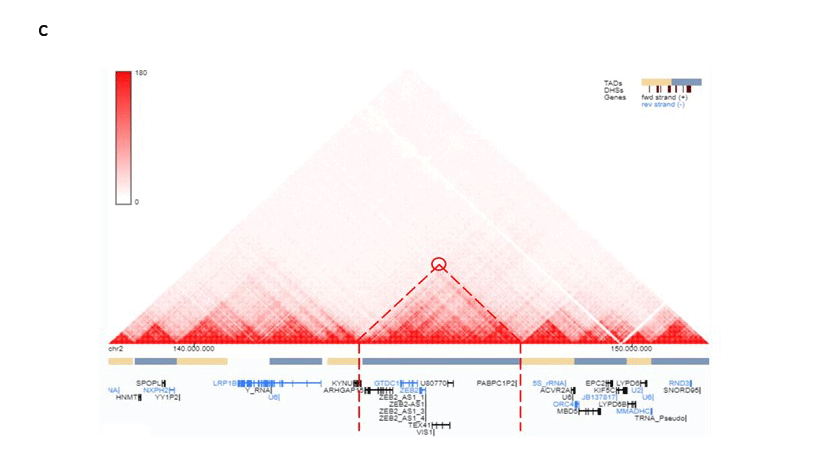
**

**Figure S1**. **(A,B)** **Expression of *GTDC1*, *ZEB2* and *ARHGAP15* in the proband’s LCL.** RT-qPCR results for *GTDC1* (A), *ZEB2* and *ARHGAP15* (B) in the proband and control LCLs, showing reduced expression of *GTDC1*, as evaluated by using five primers pairs spanning from exon 5 to exon 12 of the transcript, and normal expression levels of *ZEB2* and *ARHGAP15.* Data of one representative experiment (from three independent replicates), analyzed using the 2^−ΔΔCt^ method (housekeeping gene: *GAPDH*), are shown. Statistically significant p-values were expressed as *p<0.05 and **p<0.01, respectively. ns: not statistically significant. **(C)** **View of the *GTDC1* Topologically Associated Domain (TAD)** as visualized by the 3D Genome Browser (<http://promoter.bx.psu.edu/hi-c/>). The corresponding region from the UCSC Genome Browser (hg38) was aligned underneath the heat map.

**Table S1. Primers used for the breakpoint cloning analysis (qPCR) and expression analysis (RT-qPCR)**

| **qPCR (gDNA analysis)** | | | | |
| --- | --- | --- | --- | --- |
| **Target gene** | **Probe** | **Relative amplification** | **Chromosomal coordinates (hg38)** | **Primer sequence** |
| *GTDC1* | Proximal control | 1.00 | 144118916-144119017 | F: TGGATCGTGAAAATTCCTGGGT  R: TGTGAAACCTTTATTTGGGCCA |
|  | Proximal 1 | 1.18 | 144119164-144119296 | F: GCAGGATTAATTCCCAGAGT  R: GGTTTGTGCACATGGTTAGT |
|  | Proximal 2 | 0.43 | 144119500-144119576 | F: TACCTCATACAGATTGTGCACA R: CCTTCACATTTCACCAGTCTT |
|  | Proximal 3 | 0.53 | 144120033-144120151 | F: GAAGGGTTGGAAAGAAGTCA R: GGGAAATTGGAATTGCATTC |
|  | Proximal 4 | 0.57 | 144120238-144120352 | F: TTCTGACCCACCTCACTTGC  R: TACAGATATTGGGGCAGTTG |
|  | Deletion control | 0.52 | 144150073-144150199 | F: TTCAGTAGGCCCCTCTCCTC  R: CCTCCTGAAGCCATGCAAGA |
|  | Distal 1 | 0.55 | 144193047-144193199 | F: CAGTCTCATTCCACCACAGGT  R: GGAAAGTGGTTGAATTGCTCTC |
|  | Distal 2 | 0.49 | 144193994-144194075 | F: ACATTATGGTTTCGGGCCAA  R: TGTGAGTAGGAGAGATGACATAGA |
|  | Distal 3 | 1.22 | 144194676-144194821 | F: CGGAAGGCATTCTTGGTTAG  R: GCCATCTGAATCAGTATTTCCA |
|  | Distal 4 | 1.24 | 144195234-144195334 | F: GTTTCTGGAACAGGAGAAGG  R: GGTGAGTACCAGCCTCTTAGAA |
|  | Distal control | 1.06 | 144195495-144195654 | F: CACTACCATCTGCCAGCATT R: GGAAAATGCACATACGTACACA |
| **RT-qPCR (cDNA analysis)** | | | | |
| **Target gene** | **Probe** | **Relative amplification** | **Transcript coordinates** | **Primer sequence** |
| *GTDC1* | ex 5-6 | 0.49 | NM_001376312.2: c.456_579 | F: CCTGACCTTGGGAAACTGAAA  R: CCACCAGGCATGAAAGAATTTG |
|  | ex 6-7 | 0.52 | NM_001376312.2: c.713_811 | F: TTACTTTCCCATCAGGTTTCC  R: GCACCGCCATTTCCTTTA |
|  | ex 7-8 | 0.68 | NM_001376312.2: c.981_1124 | F: GATAATTCAAGCTCTCATCATGG R: GCTTTCTGGATCTTTATCATGC |
|  | ex 9-10 | 0.37 | NM_001376312.2: c.1262_1375 | F: CTACTTACCCAGCAAAGATGACTAT  R: CCACAGTACACGCTTCCAA |
|  | ex 10-12 | 0.14 | NM_001376312.2: c.1376_1531 | F: GTGTTACCCACTTTGTCCTAA  R: GGAGCGATTTCACCCTTATAG |
| *ZEB2* | ex 3-5 | 1.04 | NM_014795.4: c.571_670 | F: AGATGGTCCAGAAGAAATGAAG  R: GCAATTTGCATTCTTCACTGTA |
| *ARHGAP15* | ex 10-12 | 0.98 | NM_018460.4: c.983_1059 | F: AAGAGGTCTAGATGTTGATGGAAT  R: CCAAATTCAGCTTCTCTTCTTG |

**Table S2. List of up-regulated genes in the proband’s LCL (n=74)**

| **Up-regulated genes** |
| --- |
| *ANO5, ASRGL1, BIRC3,* ***CCL17****,* ***CCL22****,* ***CCL3L1****,* ***CCL4L1****,* ***CCR8****, CD83, CDCP1, CNKSR3, CNN3, DMKN, DSG2, FABP3, FHL1, GLYATL2, GTF2H2, GTSF1, HERC5, HIC1, HIST1H1C, HIST1H1D, HIST1H2AE, HIST1H2AH, HIST1H2AI, HIST1H2AJ, HIST1H2BG, HIST1H3C, HIST1H3D, HIST1H3G, HIST1H4A, HIST1H4B, HIST1H4D, HIST1H4E, HIST1H4H,* ***HLA-DRB4****,* ***HLA-DRB5****, HOMER1, IFIT3, INSIG1, ISG15, LAPTM4B, LGALS3BP, LOC102724219, LOC102724843, LOC102724951, ME1, MMP7, MOCOS, MSX1, MYO1B,* ***NFKBIA****, NID1, NOMO3, OXTR,* ***PAG1****, PEG10, PLA2G16, PNMA8A, RAB31, RPSAP58, SERPINB10, SLC45A3, SLFN11, SOX9, SSTR2, SYNGR1, TBC1D3,TC2N, TNFRSF21, TUBB2A, TUBB2B, UBD, USP18* |

Genes belonging to the “chemokine-mediated signaling” and “phosphorylation of CD3 and TCR zeta (ζ) chains” pathways are respectively colored in blue and purple.

**Table S3. List of down-regulated genes in the proband’s LCL (n=132)**

| **Down-regulated genes** |
| --- |
| *ACY3, AIF1,* ***ALDH2****,* ***ALDH7A1****, APELA, ARHGEF11, BASP1, BEX4, BHLHE40, BLK, C10orf99, C11orf74, C16orf74, CCL3L3, CCL4L2, CD99, CDIP1, CDK2AP2, CDKN1A, CHI3L2, CKLF, CORO1B, CTNND1, CTSW, CXCR3, CXXC1, CYTH4, DERL3, DOK1, DSTN, DUSP23, ECHDC2, EGR1, ESAM, FAM129B, FAM24B, FCER1G, FES, FLOT2, FUT7, FXYD2, FYN,* ***GAMT****, GDPD5, GGT1, GIMAP6,* ***GLDC****,* ***GPAT2****, GPC4, GPR18, GPR55, GSN, GSTM2, GSTM4, GTDC1, HEBP2, HLA-DRB3, ID3, IFITM3, IGLL5, IL10, IRAK3, ITGB5, ITGB7, ITM2C, KRT18, LARGE1, LARP6, LAT, LGALS1, LGALS3, LOC102723360, LPAR6,* ***LPIN1****, MACROD2, MGST2, MOXD1, MYO1F, NLRP2, NRGN, NT5E, P2RX1, PALD1, PCGF2, PCSK6, PIGR, PKIG, POLR2J3, PRKCH, PTGR1, PTMS, PTPRJ, PVRIG, PXN, RAMP1, RASA4, RGS2, RIMKLB, RNASE6, RUBCNL, S100A4, SCIMP, SGK1, SLC12A7, SLC12A8, SLFN13, SMAD1, SMCO4, SOCS3, SORBS3, SPINT2, STEAP1, SULT1A1, TBC1D3L, TBC1D4, TBX15, TBX21, TBXAS1, TCL1A, TIMP1, TMEM173, TOX, TOX2, TPM2, TRAM2, U2AF1L5, UPK3BL1, WFDC2, WWC3, ZFP36, ZNF205, ZNF300* |

Genes belonging to the “glycine, serine and threonine metabolism” and glycerolipid metabolism” pathways are respectively colored in blue and purple. The double color used for *ALDH7A1* indicates its involvement in both pathways.
